# An unexpected cause of lower gastrointestinal bleeding

**DOI:** 10.1002/ccr3.3549

**Published:** 2020-11-20

**Authors:** Daisuke Murakami, Hideaki Harada, Yuji Amano

**Affiliations:** ^1^ Department of Gastroenterology New Tokyo Hospital Chiba Japan; ^2^ Department of Endoscopy New Tokyo Hospital Chiba Japan

**Keywords:** lower gastrointestinal bleeding, urgent colonoscopy, water immersion

## Abstract

Although appendiceal bleeding is rare, physicians should keep this finding in mind as a possible cause of bleeding from the right colon because the colonoscopic diagnosis may be essential in determining the need for surgical intervention.

## Graphical abstract

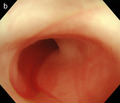

## 1 INTRODUCTION

Appendiceal bleeding is a rare cause of gastrointestinal bleeding; however, surgical appendectomy is generally recommended after a colonoscopic diagnosis. Therefore, physicians should keep this finding in mind as one of the possible causes of bleeding from the right colon and in deciding to perform a colonoscopy urgently.

**Figure 1:**
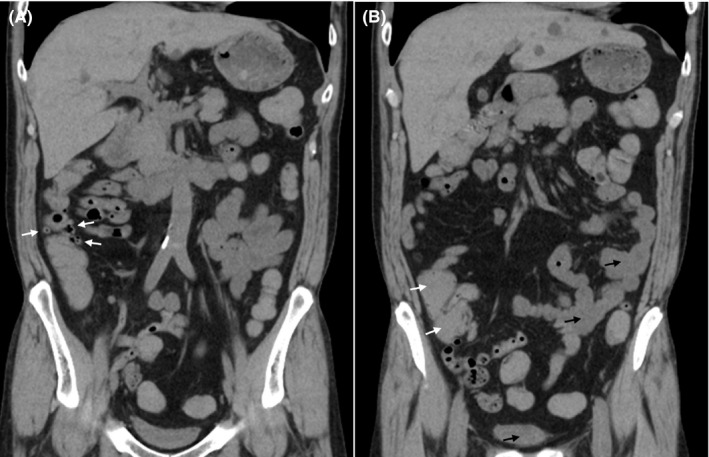
A, Nonenhanced computed tomography showed the presence of diverticula (white arrow), as gas‐filled outpouchings from the ascending colon. B, CT revealed high‐density contents in right colon (white arrow) compared with the small bowel and rectum (black arrow): the CT values in the cecum were more than twice the small intestine and rectum CT values

**Figure 2:**
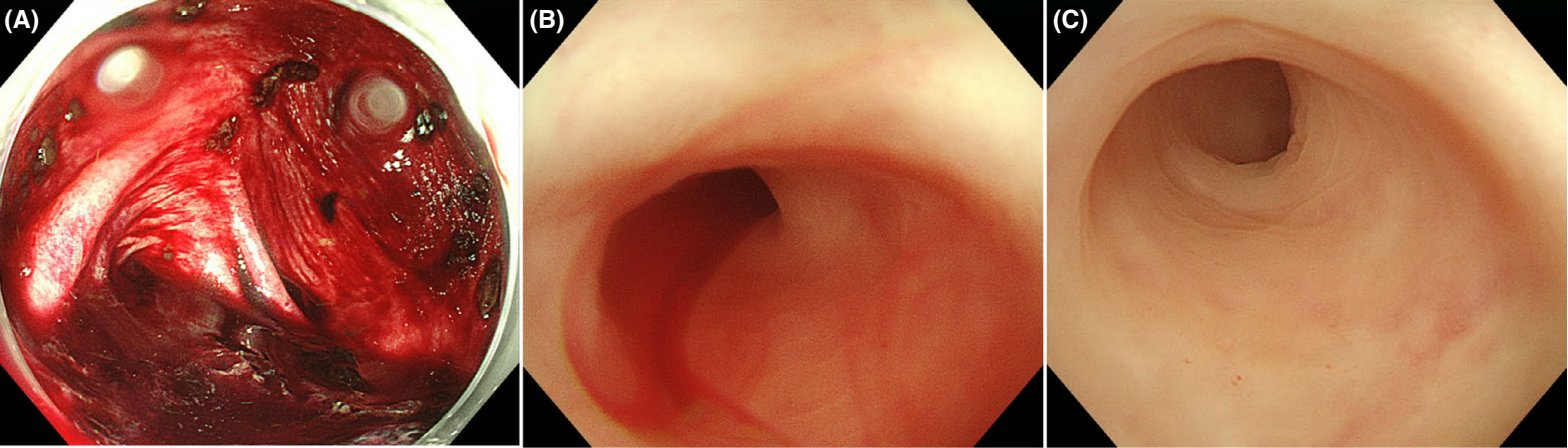
A, Urgent colonoscopy showed fresh clots in the cecum near the appendiceal orifice. B, Active intraluminal bleeding from the appendiceal orifice was confirmed by use of the water immersion method, that is, a blood stream was noted in the water‐filled lumen. C, The source of hemorrhage was not identified in spite of deep intraluminal observation of the appendix with continuous flow of water; endoscopic hemostasis was not achieved

A 54‐year‐old man with a benign medical history sought evaluation in our emergency department for a painless, massive, bloody stool. His vital signs were normal, except for tachycardia. Computed tomography showed diverticula and high‐density contents in the ascending colon and cecum (Figure 1A, B). Diverticular bleeding was suspected, and a colonoscopy was performed emergently. The colonoscopy revealed an abundance of fresh clots in the cecum near the appendiceal orifice (Figure 2A). Persistent bleeding from the appendiceal orifice was confirmed with water immersion,^1^ but the source of bleeding was not identified and endoscopic hemostasis was not achieved (Figure 2B, C). An emergency appendectomy was performed, after which the bleeding stopped. Pathologic evaluation of the specimen did not demonstrate the source of appendiceal bleeding, such as erosions, angiodysplasia, inflammation, or tumors. He has not used anticoagulants or analgesics on a regular basis; therefore, the lower gastrointestinal bleeding (LGIB) was diagnosed as idiopathic appendiceal bleeding.

The management of LGIB is usually conservative because the bleeding stops spontaneously in most cases. Despite the rarity, appendiceal bleeding is difficult to recognize using diagnostic modalities other than colonoscopy.^2^ Thus, physicians should keep this disease in mind as one of the possible causes of LGIB from the right colon because the colonoscopic diagnosis may be essential in determining the need for surgical intervention.

## CONFLICT OF INTEREST

None declared.

## AUTHOR CONTRIBUTIONS

DM: involved in study conception, acquisition of data, and drafting of the article. DM, HH, and YA: involved in analysis and interpretation of data. YA: involved in critical revision of the article for important intellectual content and final approval of the article. DM: agreed to be accountable for all aspects of this work.

## ETHICS STATEMENT

This article is the authors' own original work, which has not been previously published elsewhere and not currently being considered for publication elsewhere. All sources used are properly disclosed. All authors have been personally and actively involved in substantial work leading to the paper and will take public responsibility for its content. We certify that the procedures were performed in accordance with the ethical standards of the responsible committee on human experimentation (Institutional Review Board) and the modified Declaration of Helsinki in 2000, as well as national law. Informed consent or substitute for it was obtained from the patient for being included in the study. We declare that this submission follows the policies of Wiley's Ethics Guidelines.

## Data Availability

The authors confirm that the data supporting the findings of this study are available within the article.
